# The Impact of Exercise on Improving Body Composition and PSA in High-Risk Prostate Cancer Patients on Androgen-Deprivation Therapy

**DOI:** 10.3390/nu14235088

**Published:** 2022-11-30

**Authors:** Yu-Ching Lin, I-Hung Shao, Yu-Hsiang Juan, Kun-Yun Yeh, Chen-Pang Hou, Chien-Lun Chen, Kai-Jie Yu, Liang-Sien Chen, Chin-Li Lin, Hai-Hua Chuang

**Affiliations:** 1 Department of Medical Imaging and Intervention, Chang Gung Memorial Hospital at Keelung, Keelung 204, Taiwan; 2 College of Medicine, Chang Gung University, Taoyuan 333, Taiwan; 3 Division of Urology, Department of Surgery, Chang Gung Memorial Hospital at Linkou, Taoyuan 333, Taiwan; 4 Graduate Institute of Clinical Medical Sciences, College of Medicine, Chang Gung University, Taoyuan 333, Taiwan; 5 Department of Medical Imaging and Intervention, Chang Gung Memorial Hospital at Taoyuan, Taoyuan 333, Taiwan; 6 Division of Hemato-Oncology, Department of Internal Medicine, Chang Gung Memorial Hospital, Keelung 204, Taiwan; 7 Department of Healthcare Management, Yuanpei University of Medical Technology, Hsinchu 300, Taiwan; 8 Department of Family Medicine, Chang-Gung Memorial Hospital, Linkou Branch, Taoyuan 333, Taiwan; 9 Department of Athletics Training and Health, College of Exercise and Health Sciences, National Taiwan Sport University, Taoyuan 333, Taiwan; 10 School of Medicine, National Tsing Hua University, Hsinchu 300, Taiwan; 11 Department of Industrial Engineering and Management, National Taipei University of Technology, Taipei 106, Taiwan

**Keywords:** androgen deprivation therapy, body composition, exercise, high-risk prostate cancer, prostate-specific antigen

## Abstract

This prospective study investigated how exercise impacted chronological changes in anthropometrics, body composition, prostate-specific antigen (PSA) level and prognostic nutrition index (PNI) in high-risk prostate cancer (PCa) patients on androgen deprivation therapy (ADT). The patients were divided into either the usual care or exercise group. All patients received measurements a week before ADT initiation, six- and twelve months after treatment. The exercise group received both aerobic and resistance training. The analysis was conducted using appropriate statistical methods. There were 45 males enrolled (age 67.4 ± 8 years and BMI 25.5 ± 3.6 kg/m^2^). Profound changes were observed at six months follow-up. The exercise group showed a significant increase in the trunk and leg lean mass, and a lesser loss of total and arm lean mass. A significant decrease in PSA was also observed among the exercise group. PNI and PSA were significantly associated with regional lean mass. Exercise can prevent loss or even increase lean mass in high-risk PCa, especially in the early stage of ADT treatment. Moreover, a strong bond between lean mass and PNI and PSA further underscores the importance of early and continuous exercise interventions.

## 1. Introduction

Prostate cancer (PCa) is the second most common cancer in men and accounts for 3.8% of all cancer deaths [1,2]. Fortunately, androgen deprivation therapy (ADT), alone or as an adjuvant therapy, is able to control the growth of PCa by reducing the production of testosterone and has been the backbone treatment for advanced PCa [3,4,5]. However, ADT influences body composition negatively [6,7]. ADT can increase fat mass and decrease lean mass, leading to osteoporosis, sarcopenia, obesity, metabolic syndrome and cardiometabolic disorders [8,9]. Unfavorable body composition alterations are associated with poor prognosis in PCa patients [10,11]. It was reported that sarcopenia was associated with neutropenia, and high visceral fat volume was associated with reduced survival in castration resistant PCa patients [10]. Moreover, bone mineral density and muscle mass were independent predictors of noncancer death in PCa [11].

Many recent studies have demonstrated that exercise, aerobic and resistance, improves physical functioning, body composition, and PSA level in patients receiving ADT treatments [12]. According to previous literature, exercise may improve whole-body lean tissue mass estimates to 1 kg [7,13] and may reduce whole-body fat mass estimates to 0.66% [13]. Moreover, the difference in total fat mass between the usual care and the exercise group may reach up to 1.4 kg [6]. In addition, high-intensity interval training may decrease PSA levels to around −1.1 μg/L in three months [12]. On the contrary, some studies have discrepant results and reported no significant changes in body composition [7] and PSA level after exercise intervention [14,15]. Moreover, a weak link between body composition and PSA was also reported [14].

The major obstacle to reaching a coherent result across different studies may be related to diverse demographic characteristics, heterogeneous clinical staging, and disparate treatment modalities among studies. Thus, we conducted a prospective cohort study of patients with high-risk PCa. The enrolled patients were stage ≥ III patients who received standard ADT at a single institution to minimize bias effects from a heterogeneous population and different treatment protocols. High-risk PCa has a higher relapse rate and lower survival rates [11] and is more susceptible to deleterious effects on body composition. Thus, we hypothesize that implementing aerobic and resistance exercise into ADT treatment might have a profound effect on high-risk PCa patients’ body composition, PSA level, and nutrition status. However, literature concerning the impact of excise on the body composition among high-risk PCa patients is still limited. To understand the importance of exercise on high-risk PCa patients, we will first compare the body composition difference between the usual and exercise group during 6 and 12 months of ADT treatment. Furthermore, we will also compare the PSA and nutrition index differences in those two groups to understand the impact of excise on the PSA level and nutrition. We believe that this study will demonstrate the importance of exercise in improving treatment outcomes of high-risk PCa patients.

This study first aimed to investigate the chronological body composition change in high-risk PCa in both usual and exercise groups and secondly, to investigate the association of body composition with PSA level and nutrition.

## 2. Materials and Methods

### 2.1. Patients Enrollment

This prospective cohort study was performed between May 2019 and November 2021. This study was approved by the institutional review board (approval number: 201702135B0, 201801389A3C103) and was performed by the good clinical practice guidelines and the Declaration of Helsinki. Written informed consent was obtained from all patients.

Eligible patients were aged 20–80 years with histologically proven prostatic adenocarcinoma and classified as high-risk PCa. High-risk PCa was classified according to the Radiation Therapy Oncology Group classification, including (1) Gleason ≥ 8, or (2) Gleason = 7 plus either ≥ cT3 or node-positive [16].

According to the seventh edition of the American Joint Committee on Cancer (AJCC) staging system, clinical tumor staging was conducted by combining enhanced CT, magnetic resonance imaging, PSA level, and Gleason scores. All eligible patients will receive standard ADT set by our hospital and regular computed tomography (CT) image follow-up. Patients were excluded if they had lost follow-up, had end-stage renal failure, were allergic to contrast medium, had an ongoing infection, and received regular medications that could substantially modulate metabolism or weight, such as steroids or megestrol acetate. A total of 55 eligible patients were recruited, among whom 10 patients lost follow-up and were excluded from this study.

### 2.2. Androgen Deprivation Therapy Protocol

ADT was carried out either by surgical castration or chemical castration. The decision of surgical or chemical castration was decided based on each clinical scenario after discussion with the patients. Surgical castration was performed with simple orchiectomy under spinal or general anesthesia. Chemical castration was achieved with Gonadotropin-Releasing Hormone (GnRH) agonist or antagonist. The dose, interval, and injection methods (subcutaneous or intramuscular) were based on manufacturers’ suggestions. After ADT is performed, serum testosterone will be checked to ensure the castration level was achieved (<50 ng/dL).

### 2.3. Exercise Program

The patients were allocated randomly to either the exercise or the usual care group in a 2:1 ratio. The exercise group was asked to complete a 24-week exercise program. The usual care group maintained their usual exercise levels.

The exercise program was designed by a multidisciplinary team experienced in physical fitness promotion [17,18], consisting of certified personal trainers, sport science experts, and family medicine physicians. Several urological oncologists also participated in the development of the exercise protocol. The program was initiated within one week after the first ADT dose and lasted for 24 weeks. Each patient was asked to complete at least two nonconsecutive sessions per week, and 60 min per session. Each session was structured, including 20 min of warm-up and aerobic exercise, 30 min of resistance training, and 10 min of cool-down. The volume and intensity were moved forward slowly with cautions according to each patient’s baseline fitness and pre-existed medical conditions.

Each patient in the exercise group had a minimum of three one-on-one instruction sessions with a certified personal trainer at the investigation site during the 24 weeks. The patients could choose to take the rest of the sessions either physically onsite or remotely at home for infection control during the COVID-19 pandemic.

### 2.4. Clinicopathological Data

Clinicopathological data were collected, including age, body mass index (BMI), smoking history, alcohol consumption, and betel nut consumption. The comorbid diseases, including hypertension, cerebrovascular accident, and diabetes mellitus, were recorded. Surgical treatment (radical prostatectomy), Gleason score, and AJCC 7th edition of tumor stage.

### 2.5. Body Composition Measurement

Body composition measurements were obtained using two different imaging modalities, dual-energy fan-beam X-ray absorptiometry (DXA) and CT. DXA provided body composition in different anatomical regions, whilst CT provided a more detailed quantification of core muscles and abdominal fat.

Total body composition was measured using the Hologic Horizon DXA system (Hologic Inc., Bedford, MA, USA) with array scan mode following each manufacturer’s protocol for body composition measures. Scans were analyzed using Hologic APEX Software, version 5.6.0.4. Each participant was positioned according to the guidelines set by the International Society for Clinical Densitometry [19]. DXA was used to acquire the lean and fat mass of the different anatomical regions, including the arms, legs, and trunk. Appendicular skeletal muscle index (ASMI) was also calculated by summing the bilateral arm lean mass with the bilateral leg lean mass divided by height in square meters.

In addition to DXA, specific core muscles, including psoas and paraspinal (consisting of quadratus lumborum, erector spine muscles) muscles and abdominal fat, including subcutaneous adipose tissue (SAT) and visceral adipose tissue (VAT), were quantified by unenhanced abdominal CT scan. The DXA scan was only able to provide the overall trunk region measurement but not a specific region of muscle and fat. However, improving the core muscle, SAT, and VAT is essential in aerobic and resistance exercise and should also be monitored. Thus, we measured core muscle, SAT, and VAT using a CT scan. Core muscle was measured at L3 level abdomen with 5 mm slice thickness. Four adjacent axial images selected from the L3 level were measured and averaged for final analysis [20]. The following data were acquired: total cross-section area (cm^2^) and the selected region’s mean density/Hounsfield unit (HU).

### 2.6. Handgrip Strength Assessment

Muscle strength was assessed using dynamometers (EH101; Camry, Zhongshan, China) to measure the handgrip strength (HS). Three measures on the dominant hand were taken, and the maximum was recorded.

### 2.7. Laboratory Examinations

The results of all laboratory examinations were obtained and processed by a centralized laboratory in our institution. Four different laboratory parameters were assessed. The nutrition status of the patients was evaluated by cholesterol level and prognostic nutrition index (PNI). PNI was calculated as 10 × serum albumin (g/dL) + 0.005 × total lymphocyte count (/mm^3^); the inflammatory status was evaluated by the ratio of the platelet count to lymphocyte count (PLR); the tumor response to ADT was assessed by the level of PSA.

All patients received an imaging study, HS assessment, and laboratory examination a week before ADT initiation, six- and twelve months after treatment.

### 2.8. Statistical Analysis

The demographic distribution of the exercise and usual groups was compared, using an independent sample t-test for continuous variables and Fisher’s exact test for categorical variables. The chronological change of body composition (DXA and CT analysis), hand grip strength, and laboratory parameters (PNI, PLR, and PSA) between the exercise and the usual groups from baseline to sixth and 12th months was compared using a generalized estimating equation (GEE) with exchangeable working correlation matrix. The GEE model included intercept, main effects of time points and study group (exercise vs. usual) and the interaction effects between time points and study group. The change value between groups was considered different when the interaction effect was significant. In addition, the GEE adjusted for baseline BMI and total lean since there were borderline significant differences between the two groups (see Table 1). Furthermore, the association between a change in body composition (e.g., DXA, CT parameters and muscle function) and a change in PNI and PSA was evaluated using linear regression analysis. All tests were two-tailed and *p* < 0.05 was considered statistically significant. Data analyses were conducted using SPSS 26 (IBM SPSS Inc., Chicago, IL, USA).

## 3. Results

### 3.1. Baseline Demographics

The baseline demographics are listed in Table 1. There were forty-five males (mean age was 67.4 ± 8 years) eligible for this study. The majority of patients comprised stage 4B (44.4%) and followed by stage 3B (28.9%), stage 4A (17.8%), and stage 3C (8.9%). The mean BMI was within the normal range (25.5 ± 3.6 kg/m^2^) with a mean total body fat of 23,005.4 ± 6661.6 g and a mean total lean mass of 43,552.6 ± 4881.8 g. There were 31 and 14 subjects in the exercise and usual groups, respectively. The baseline BMI and total lean values were borderline significantly lower in the exercise group (*p* = 0.052 and 0.099). Otherwise, no significant difference in the baseline demographics between groups was noted.

### 3.2. Comparison between the Exercise and Usual Care Group before, during, and after Intervention

The comparison of DXA, CT, and laboratory parameters between the exercise and usual care group at baseline, the sixth month and the 12th month is shown in Table 2, Table 3 and Table 4.

#### 3.2.1. At Six Months Follow-Up

There was a significant increase in BMI by 3.19% in the exercise group when compared to the usual group (mean difference [MD] = 1.4, 95% confidence interval [CI] = 0.44 to 2.35). The above was contributed by the significant increase in lean mass in the trunk (0.90%), leg (1.88%) and ASMI (0.78%) and a significant increase in visceral fat area (14.09%) in the exercise group. Moreover, there was a lesser decrease in arm lean mass (−1.88% vs. −6.87%, MD = 232.03, 95% CI = 1.97 to 462.10), and total lean mass (0.79% vs. −12.21%, MD = 5666, 95% CI = 1547 to 9785) when the exercise group and the usual group were compared. Notably, there was a significant increase in handgrip strength (1.41%) in the exercise group when compared to the usual group (−9.25%) (Table 3). In the DXA measurement, the most noticeable improvement was found in lean mass rather than fat in the exercise group. Moreover, the degree of improvement was discrepant in different regions of interest. Even though the fat change was not apparent in the DXA measurement, a detailed CT measurement still noted a significant increase in the visceral fat area. In addition, there was a significant decrease in the PSA level (−102.62% vs. −64.43%, MD = −223.97, 95% CI = −443.43 to −13.51) and a borderline significant lesser degree of decrease in PNI (−3.61% vs. −9.74%, MD = 3.48, 95% CI = −0.12 to 7.09) when the exercise group and the usual group were compared (Table 4). The exercise group improves lean mass, muscle function, and PSA level.

#### 3.2.2. At Twelve Months Follow-Up

There was a borderline significant increase in BMI in the exercise group when compared to the usual group (4.18%), which mainly contributed to the increasing trunk lean mass (0.61%) and visceral fat area (18.03%) (Table 2 and Table 3). Notably, there was a decrease in trunk lean mass in the usual group (−2.71%). However, the difference between the exercise group and the usual group in both body composition and laboratory parameters was less conspicuous after twelve months of treatment.

### 3.3. The Association between Body Composition and Laboratory Data

The potential effect of body composition on PNI and PSA at 6-month follow-up was analyzed in the exercise group and detailed in Table 5 and Table 6.

There was a significant positive association between muscle mass and function with PNI in both follow-up periods, especially in regional lean mass, para-spinal muscle area, psoas muscle area, and HS. At the 6-month follow-up, every 100 g increase in the arm, trunk and leg lean mass and ASMI were correlated with an increase of 1.79 (95% CI = 0.89 to 2.69), 0.97 (95% CI = 0.61 to 1.34), and 10.75 (95% CI = 6.93 to 14.57) units of PNI, respectively. Similarly, for every unit increase in para-spinal and the psoas muscle area, there would be an increase of 0.78 (95% CI = 0.43 to 1.13) and 0.88 (95% CI = 0.31 to 1.44) units of PNI, respectively. Moreover, for every kg increase in HS, there was an increase of 0.74 (95% CI = 0.21 to 1.26) units of PNI. Notably, there was also a significant positive association between fat tissue and PNI at 6-month follow-ups, especially in body fat mass, SAT area density, and VAT density. A strong bond was found between muscle and PNI, and the association was found across different regions of interest, including appendicular lean mass (arm and leg) and core muscle (para-spinal and psoas muscles).

As for the association of body composition and PSA, there was a significant negative association between the muscle mass and PSA in both follow-ups, especially regional lean mass, para-spinal muscle area, and psoas muscle area with PSA. At the 6-month follow-up, for every 100 g increase in arm, leg lean mass, and ASMI, there was a decrease of 107.64 (95% CI = −205.94 to −9.33), 54.72 (95% CI = −99.20 to −10.24), 555.51 (95% CI = −1040.85 to −70.18) units of PSA, respectively. Similarly, for every unit increase in para-spinal and the psoas muscle area, there was a decrease of 54.96 (95% CI = −93.00 to −16.92) and 63.06 (95% CI = −119.90 to −6.22) units of PSA, respectively. However, the association between HS and PSA did not reach statistically significant. Notably, there was a significant negative association between body fat mass and PSA in both follow-ups. Comparable results were found between PSA and PNI, and a strong bond was found between muscle and PSA. However, the only difference is that HS is not associated with PSA but with PNI.

To better understand the long-term impact of changes in body composition on the nutrition index and PSA, additional analysis on the association between the changes in body composition (from baseline to the sixth month) and changes in the prognostic nutrition index and PSA (from baseline to the 12th month) was described in Supplementary Table S1. The results were consistent with the baseline comparison.

## 4. Discussion

**Figure 1:**
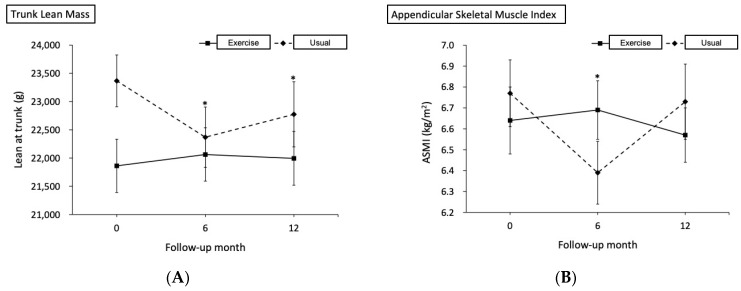

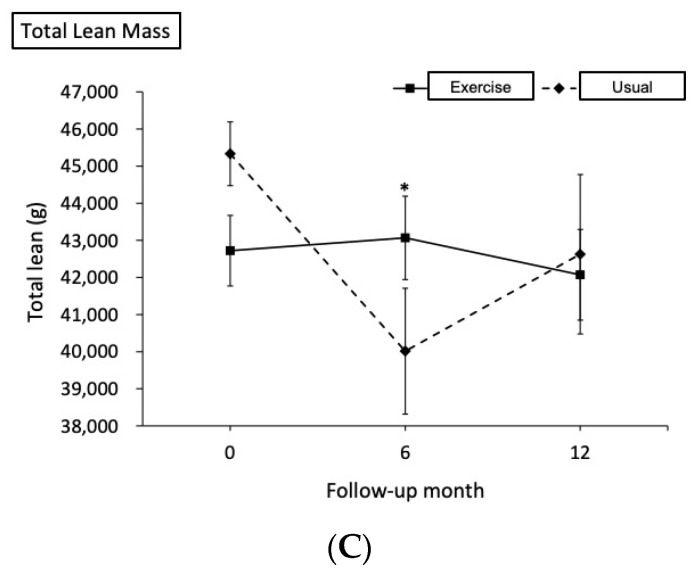
During the initial six months of androgen deprivation therapy, there was a significant increase in (**A**) trunk lean mass, (**B**) appendicular skeletal muscle index, and (**C**) total lean mass in the exercise group as compared to the usual group.

We hypothesized that exercise could mitigate the detrimental effects on body composition caused by ADT in high-risk PCa patients and subsequently lead to a decreased PSA level and improved nutrition status. Therefore, this study aimed to compare body composition change between the usual and exercise groups and understand how body composition was associated with PSA and nutrition. Our results showed a significant increase in lean mass in the exercise group after a 24-week exercise program (Figure 1). Moreover, a strong bond between regional lean mass (arm and leg) and PNI and PSA was observed. Thus, we encourage a combination exercise of both aerobic and resistance training to be carried out in high-risk PCa patients as an adjuvant therapy to ADT, since it may be beneficial for higher lean mass, lower PSA levels, and better nutrition status.

In previous reports, the commencement of an exercise program involving aerobic and resistance training when initiating ADT may significantly reduce treatment toxicity, the catabolic effect of ADT, and cancer-related fatigue [6,15,21]. The exercise may even enhance ADT treatment by promoting cytotoxic natural killer cells entering tumor cells and modulating systemic inflammatory mediators, tumor vascularization, and perfusion to suppress PCa progression [12]. Since aerobic and resistance exercise has different roles in changing body composition, the literature indicates that aerobic training is best conducted combined with resistance training [22]. Aerobic training has been suggested to be more effective in decreasing visceral fat, whilst resistance training is more likely to improve muscle mass, strength, and physical function [13,15].

Resistance training promotes protein synthesis and muscle hypertrophy via upregulating the mTOR signaling pathway [22]. Thus, we propose that resistance training was responsible for the significant increase in muscle mass (trunk lean mass and ASMI) and muscle function (HS) in the exercise group. Our results correspond to previous reports showing resistance exercise increases appendicular skeletal mass and muscle strength [23]. The maintenance of appendicular lean mass is clinically vital. Not only does ASMI serve as a diagnostic criterion for sarcopenia in the elderly [23], but this study also observed that ASMI positively impacts PSA and PNI. Thus, incorporating resistance exercise in high-risk PCa patients’ treatment may be essential to preserve the appendicular muscle mass and improve PSA and PNI.

In this group of high-risk PCa patients, the exercise group had a significantly larger drop in PSA level than the usual group at the early stage of ADT treatment (99.54% vs. 95.08%, *p* = 0.037). Jones et al. [24] demonstrated that exercise was able to modulate the expression of pro-metastatic genes and reduce metastasis in murine PCa. However, studies in humans showed inconsistent results. Galvão DA et al. [15] found no difference in PSA between the usual care and exercise groups of men with localized PCa on ADT. Segal et al. [25] found no difference in PSA levels after 24 weeks of either aerobic or resistance exercise in men with PCa receiving radiotherapy compared to the control. On the contrary, a study in Denmark reported that the PSA doubling time was significantly prolonged after a 2-year home-based endurance training [26]. We postulated that the effects of exercise differed across patient groups with various ages, disease severity, and treatment modalities. According to our data, the decline of PSA was greater in the exercise group. Our result supported our hypothesis, that the high-risk PCa patients were more susceptible to side effects of ADT, for which the beneficial effects of exercise might be more profound in this particular group.

Moreover, this study also found that exercise positively impacts the nutrition status of high-risk PCa patients. Although it was borderline significant, there was a lesser decrease in PNI in the exercise group than in the usual group at the early stage of ADT treatment (3.61% vs. 9.74%, *p* = 0.058). Patients with advanced PCa are more vulnerable to malnutrition or undernutrition due to a loss in appetite [27]. We believe that exercise can promote PCa patients’ appetite. Moreover, patients on regular exercise may also have a more positive attitude as well as a stronger self-efficacy towards a healthy diet. This study demonstrated that exercise not only reduced the PSA level but also improved the nutrition status in a sample of high-risk PCa patients. Furthermore, we provided a quantitative goal for high-risk PCa patients. For every 100 g increase in ASMI, there would be a decrease of 555.51 units of PSA and an increase of 10.75 units of PNI, respectively. Setting a quantitative goal helps the clinician encourage the patient to exercise, and patients might be more enthusiastic about exercise.

Of note, other than multiple lean-mass-related parameters, the exercise group also had a higher VAT area in both the sixth and 12th follow-ups. It is evident that low lean mass is an independent predictor for worse prognosis including treatment-related toxicities, surgical complications, risk of recurrence, cancer-specific mortality, and overall survival [28]. Nonetheless, a considerable volume of literature also suggests that fat tissue may have some kind of protective effect for some cancer patients. A systematic review reported a significant association between higher SAT and higher survival in men with PCa. [29] Charette et al. demonstrated that colorectal cancer patients with low SAT and VAT index had an increased risk of dying, while high VAT density was a predictor of poor survival. The role of adiposity in the survival of cancer patients remains inconclusive. Subcutaneous and visceral fat not only differ in anatomical location, but they are also very different in tissue structures, metabolic functions, and inflammatory tendencies. We proposed that since exercise might lead to a more anabolic status, the patients in the exercise group gained more weight than the usual group mostly in lean mass but also partially in fat. Future investigations on the body composition of PCa patients are warranted to further clarify how types, quantity and quality of adipose tissue impact treatment response and survival.

Last but not least, almost all drastic changes, including in lean mass, PSA, and PNI, were observed only in the sixth month but not in the 12th month, suggesting that after the completion of the exercise program, the effects of exercise waned over 6 months. This finding further highlights the importance of an early and continuous exercise intervention in high-risk PCa to prevent or ease the side effects of ADT. The development of ADT toxicity and adverse effects are most prominent in the first six months of treatment [6,13,21]. Reducing ADT toxicity and adverse effects will definitely increase the patients’ willingness to complete ADT treatment and improve the clinical outcome of high-risk PCa. Thus, we encourage the combination of aerobic and resistance exercise to be carried out in high-risk PCa as an adjuvant therapy to ADT treatment as early as possible.

There were some limitations that deserve mention. First, the study sample consisted of homogenous high-risk PCa patients; however, the sample size was not as large as we expected due to the overlapping of our enrollment period and the pandemic of COVID-19. Second, there was an uneven grouping of the exercise and the usual group because of the 2:1 subject allocation method. Third, this study used PSA level as a parameter for treatment response, which might not be sufficient to represent long-term treatment outcomes. Studies with longer follow-up times and data on survival duration or mortality will be of interest in the future. Fourth, about half of this study population received radical prostatectomy. Still, the literature concerning the effect radical prostatectomy has on body composition is limited. Therefore, we believe that radical prostatectomy’s influence on body composition should be minor compared to laparostomy.

## 5. Conclusions

The combination of aerobic and resistance exercise positively impacted body composition, PSA, and PNI in high-risk PCa patients. Exercise had more influence on lean mass than fat mass. It improves and prevents lean mass loss in the trunk and appendicular regions. Moreover, exercise can substantially decrease PSA levels and avoid loss of nutrition. Notably, the impact of exercise on lean mass, PSA, and PNI is shown at the early stage of ADT treatment and wanes after discontinuing exercise. Thus, it is crucial to initiate exercise intervention early and implement it continuously to mitigate the adverse effect of ADT treatment. In addition, there is a strong association between appendicular lean mass and PSA. For every increase of 100 g of AMSI, there is a decrease of 555.51 units of PSA. Setting a quantitative goal helps the clinician encourage the patient to exercise, and patients will be enthusiastic about exercise.

## Figures and Tables

**Table 1:** Demographics of the exercise and usual groups.

Variable	Total (*n* = 45)	Exercise (*n* = 31)	Usual (*n* = 14)	*p*-Value
Age, years	67.4 ± 8.0	66.2 ± 7.2	70.1 ± 9.3	0.128
Body mass index, kg/m^2^	25.5 ± 3.6	24.8 ± 3.7	27.1 ± 2.8	0.052
Smoking	5 (11.1)	2 (6.5)	3 (21.4)	0.166
Alcohol	7 (15.6)	3 (9.7)	4 (28.6)	0.180
Betel nut	1 (2.2)	0 (0.0)	1 (7.1)	0.311
Hypertension	21 (46.7)	13 (41.9)	8 (57.1)	0.520
Cerebrovascular accident	1 (2.2)	0 (0.0)	1 (7.1)	0.311
Diabetes mellitus	10 (22.2)	7 (22.6)	3 (21.4)	1.000
Surgery	20 (44.4)	15 (48.4)	5 (35.7)	0.525
Gleason Score	8.51 ± 0.99	8.61 ± 0.88	8.29 ± 1.20	0.311
Stage				0.677
3B	13 (28.9)	9 (29.0)	4 (28.6)	
3C	4 (8.9)	3 (9.7)	1 (7.1)	
4A	8 (17.8)	4 (12.9)	4 (28.6)	
4B	20 (44.4)	15 (48.4)	5 (35.7)	
Body composition				
Total fat, g	23,005.4 ± 6661.6	22,002.2 ± 7125.3	25,155.1 ± 5121.5	0.146
Total lean, g	43,552.6 ± 4881.8	42,721.3 ± 5301.4	45,333.9 ± 3334.8	0.099

Data are given as frequency (percentage) and mean ± standard deviation. T-test continuous variable. Fisher Exact test categoric variable.

**Table 2:** The comparison of DXA parameters between the exercise and usual groups.

Parameter/Time	Exercise (*n* = 31)		Usual (*n* = 14)		Mean Difference ^†^ (95% CI)	*p* for Interaction ^†^
	Mean (SE)	Change %	Mean (SE)	Change %		
BMI, kg/m^2^						
Baseline	25.5 (0.1)	-	25.8 (0.1)	-	-	-
6th month	26.3 (0.3)	3.19	25.2 (0.4)	−2.26	1.40 (0.44, 2.35)	0.004 *
12th month	26.5 (0.3)	4.18	25.9 (0.3)	0.72	0.88 (−0.01, 1.76)	0.052
Fat at arm, g						
Baseline	2493.6 (70.1)	-	2590.8 (80.3)	-	-	-
6th month	2756.4 (94.6)	10.54	2765.2 (81.6)	6.73	88.3 (−96.7, 273.4)	0.349
12th month	2868.3 (101.7)	15.02	2770.3 (82.4)	6.93	195.1 (−1.1, 391.4)	0.051
Fat at trunk, g						
Baseline	12,313.8 (357.2)	-	13,390.9 (496.5)	-	-	-
6th month	13,462.4 (437.9)	9.33	14,010.4 (548.3)	4.63	529.1 (−285.0, 1343.1)	0.203
12th month	13,885.0 (454.0)	12.76	14,163.1 (448.6)	5.77	799.0 (−159.8, 1757.7)	0.102
Fat at leg, g						
Baseline	6088.7 (218.1)	-	6198.0 (234.4)	-	-	-
6th month	6853.1 (253.1)	12.55	6727.3 (256.5)	8.54	235.0 (−236.2, 706.2)	0.328
12th month	7140.8 (264.7)	17.28	6948.0 (234.8)	12.10	302.0 (−171.5, 775.5)	0.211
Total fat, g						
Baseline	22,698.7 (878.7)	-	23,827.7 (711.3)	-	-	-
6th month	24,917.3 (840.2)	9.77	32,404.1 (3799.4)	35.99	−6357.7 (−13,912.3, 1196.9)	0.099
12th month	26,526.2 (1237.9)	16.86	27,518.3 (2044.6)	15.49	137.0 (−4517.4, 4791.3)	0.954
Lean at arm, g						
Baseline	4799.9 (72.9)	-	4691.9 (108.4)	-	-	-
6th month	4709.8 (82.5)	−1.88	4369.7 (129.3)	−6.87	232.03 (1.97, 462.10)	0.048 *
12th month	4632.8 (85.3)	−3.48	4430.6 (103.1)	−5.57	94.23 (−104.29, 292.75)	0.352
Lean at trunk, g						
Baseline	22,226.7 (258.5)	-	22,583.2 (268.5)	-	-	-
6th month	22,427.2 (352.2)	0.90	21,584.9 (407.3)	−4.42	1198.8 (418.2, 1979.3)	0.003 *
12th month	22,363.1 (380.7)	0.61	21,971.2 (433.3)	−2.71	748.3 (34.1, 1462.6)	0.040 *
Lean at leg, g						
Baseline	13,858.1 (170.0)	-	13,360.6 (297.3)	-	-	-
6th month	14,118.2 (215.7)	1.88	12,626.9 (377.6)	−5.49	993.8 (380.2, 1607.4)	0.002 *
12th month	13,848.3 (237.6)	−0.07	13,277.2 (361.7)	−0.62	73.7 (−687.6, 835.0)	0.850
Total lean, g						
Baseline	43,530.3 (265.1)	-	43,573.2 (436.6)	-	-	-
6th month	43,875.1 (930.1)	0.79	38,252.4 (1782.8)	−12.21	5666 (1547, 9785)	0.007 *
12th month	42,867.1 (1224.4)	−1.52	40,395.6 (2289.7)	−7.29	2514 (−2916, 7945)	0.364
Android, %Fat						
Baseline	37.6 (0.8)	-	39.2 (1.0)	-	-	-
6th month	39.3 (0.7)	4.73	41.2 (1.0)	5.16	−0.24 (−2.27, 1.78)	0.813
12th month	40.6 (0.7)	7.94	41.0 (1.1)	4.63	1.17 (−1.23, 3.56)	0.339
Gynoid, %Fat						
Baseline	30.9 (0.6)	-	31.8 (0.7)	-	-	-
6th month	33.3 (0.7)	7.75	34.5 (0.5)	8.30	−0.25 (−1.91, 1.41)	0.769
12th month	34.7 (0.7)	12.22	33.9 (0.5)	6.34	1.76 (0.002, 3.51)	0.050
ASMI, kg/m^2^						
Baseline	6.78 (0.07)	-	6.48 (0.11)	-	-	-
6th month	6.83 (0.07)	0.78	6.10 (0.12)	−5.88	0.43 (0.16, 0.71)	0.002 *
12th month	6.70 (0.09)	−1.10	6.43 (0.15)	−0.88	−0.02 (−0.35, 0.31)	0.915

Abbreviations: DXA, dual energy *x*-ray absorptiometry; CI, confidence interval; BMI, body mass index; ASMI, appendicular skeletal mass index. Data are given as estimated marginal mean (standard error). † adjusted for baseline body mass index and total lean. * denotes significant *p*-value.

**Table 3:** The comparison of Handgrip and CT parameters between the exercise and usual groups.

Parameter/Time	Exercise (*n* = 31)		Usual (*n* = 14)		Mean Difference ^†^ (95% CI)	*p* for Interaction ^†^
	Mean (SE)	Change %	Mean (SE)	Change %		
Handgrip strength, kg						
Baseline	34.64 (1.04)	-	32.08 (1.73)	-	-	-
6th month	35.12 (0.94)	1.41	29.11 (2.14)	−9.25	3.45 (0.20, 6.71)	0.038 *
12th month	34.95 (1.05)	0.91	31.88 (1.70)	−0.61	0.51 (−1.78, 2.80)	0.662
Para. area, cm^2^						
Baseline	53.26 (0.95)	-	49.75 (1.87)	-	-	-
6th month	52.57 (1.20)	−1.29	46.96 (1.72)	−5.62	2.11 (−0.61, 4.83)	0.128
12th month	52.98 (1.18)	−0.53	47.31 (1.79)	−4.92	2.16 (−1.40, 5.73)	0.235
Para. density, HU						
Baseline	38.81 (1.44)	-	37.33 (2.11)	-	-	-
6th month	38.00 (1.56)	−2.08	33.41 (2.13)	−10.51	3.12 (0.88, 5.35)	0.006 *
12th month	35.40 (1.56)	−8.80	30.80 (2.90)	−17.49	3.12 (−1.01, 7.24)	0.139
Ps. area, cm^2^						
Baseline	16.79 (0.67)	-	14.42 (0.75)	-	-	-
6th month	15.17 (0.62)	−9.67	12.50 (0.95)	−13.35	0.30 (−1.40, 2.00)	0.728
12th month	14.87 (0.51)	−11.47	13.13 (0.81)	−8.98	−0.63 (−2.47, 1.20)	0.500
Ps. density, HU						
Baseline	42.88 (0.94)	-	43.39 (2.17)	-	-	-
6th month	42.45 (0.77)	−1.00	44.50 (3.78)	2.56	−1.54 (−7.80, 4.72)	0.629
12th month	41.54 (0.80)	−3.13	43.44 (3.71)	0.13	−1.40 (−8.86, 6.06)	0.713
SAT area, cm^2^						
Baseline	116.77 (5.62)	-	122.47 (6.75)	-	-	-
6th month	133.76 (8.09)	14.56	131.35 (6.87)	7.25	8.11 (−5.72, 21.95)	0.250
12th month	140.42 (7.86)	20.26	132.96 (8.19)	8.57	13.16 (−5.75, 32.08)	0.173
SAT density, HU						
Baseline	−87.97 (2.14)	-	−90.28 (1.55)	-	-	-
6th month	−91.86 (1.20)	4.42	−92.50 (1.38)	2.46	−1.67 (−6.17, 2.83)	0.467
12th month	−93.45 (1.44)	6.23	−90.92 (1.70)	0.72	−4.83 (−11.09, 1.42)	0.130
VAT area, cm^2^						
Baseline	172.23 (9.37)	-	202.89 (17.66)	-	-	-
6th month	196.49 (10.87)	14.09	202.42 (18.04)	−0.23	24.73 (3.75, 45.71)	0.021 *
12th month	203.28 (12.13)	18.03	206.02 (19.71)	1.54	27.93 (3.26, 52.59)	0.026 *
VAT density, HU						
Baseline	−92.96 (1.72)	-	−95.04 (1.21)	-	-	-
6th month	−94.62 (1.16)	1.79	−95.37 (1.55)	0.35	−1.34 (−4.71, 2.04)	0.438
12th month	−96.77 (1.25)	4.10	−93.46 (1.74)	−1.66	−5.39 (−10.36, −0.42)	0.034 *

Abbreviations: CI, confidence interval; Para., paraspinal muscle; Ps., psoas muscle; SAT, subcutaneous adipose tissue; VAT, visceral adipose tissue. Data are given as estimated marginal mean (standard error). † adjusted for baseline body mass index and total lean. * denotes significant *p*-value.

**Table 4:** The comparison of laboratory examinations between the exercise and usual groups.

Parameter/Time	Exercise (*n* = 31)		Usual (*n* = 14)		Mean Difference (95% CI) ^†^	*p* for Interaction ^†^
	Mean (SE)	Change %	Mean (SE)	Change %		
Prognostic nutrition index						
Baseline	53.63 (0.78)	-	55.65 (1.09)	-	-	-
6th month	51.70 (0.89)	−3.61	50.23 (1.54)	−9.74	3.48 (−0.12, 7.09)	0.058
12th month	50.58 (0.58)	−5.70	50.38 (1.61)	−9.47	2.21 (−0.93, 5.36)	0.168
Platelet lymphocyte ratio						
Baseline	150.15 (12.32)	-	112.76 (11.13)	-	-	-
6th month	180.92 (14.73)	20.49	217.18 (37.46)	92.60	−73.65 (−157.32, 10.02)	0.084
12th month	190.46 (19.60)	26.85	169.12 (29.29)	49.98	−16.05 (−82.84, 50.74)	0.638
Total cholesterol, mg/dL						
Baseline	180.80 (7.10)	-	210.85 (9.44)	-	-	-
6th month	193.94 (8.20)	7.26	212.78 (15.44)	0.91	11.20 (−16.44, 38.85)	0.427
12th month	189.53 (7.36)	4.83	211.87 (15.92)	0.48	7.71 (−24.61, 40.03)	0.640
PSA, ng/mL						
Baseline	247.86 (104.34)	-	47.16 (13.98)	-	-	-
6th month	−6.50 (8.10)	−102.62	16.77 (9.33)	−64.43	−223.97 (−434.43, −13.51)	0.037 *
12th month	7.51 (15.65)	−96.97	26.42 (11.36)	−43.99	−219.60 (−437.25, −1.94)	0.048 *

Abbreviations: CI, confidence interval; PSA, prostate-specific antigen; Data are given as estimated marginal mean (standard error); † adjusted for baseline body mass index and total lean. * denotes significant *p*-value.

**Table 5:** The association between change of body composition and change of prognostic nutrition index in the exercise group.

Parameters	6th Month—Baseline		12th Month—Baseline	
	*B* (95% CI)	*p*-Value	*B* (95% CI)	*p*-Value
**DXA parameters**				
Fat, per 100 g				
Arm	1.48 (0.41, 2.55)	0.008 *	0.47 (0.07, 0.88)	0.023 *
Trunk	0.18 (0.06, 0.29)	0.004 *	0.12 (0.04, 0.20)	0.007 *
Leg	0.76 (0.35, 1.18)	0.001 *	0.23 (0.08, 0.38)	0.004 *
Total	0.32 (−0.10, 0.75)	0.129	0.10 (−0.17, 0.37)	0.448
Lean, per 100 g				
Arm	1.79 (0.89, 2.69)	<0.001 *	0.57 (0.19, 0.95)	0.005 *
Trunk	0.002 (−0.48, 0.48)	0.994	−0.31 (−0.65, 0.03)	0.073
Leg	0.97 (0.61, 1.34)	<0.001 *	0.21 (0.08, 0.34)	0.002 *
Total	−0.08 (−0.31, 0.16)	0.514	−0.13 (−0.31, 0.05)	0.137
ASMI, kg/m²	10.75 (6.93, 14.57)	<0.001 *	4.44 (1.58, 7.29)	0.004 *
**CT parameters**				
SAT				
Area, cm^2^	0.12 (0.06, 0.19)	0.001 *	0.08 (0.03, 0.13)	0.004 *
Density, HU	−0.33 (−0.50, −0.15)	0.001 *	−0.11 (−0.23, 0.01)	0.081
VAT				
Area, cm^2^	0.05 (−0.01, 0.12)	0.095	0.05 (0.01, 0.09)	0.015 *
Density, HU	−0.53 (−0.72, −0.33)	<0.001 *	−0.21 (−0.34, −0.07)	0.004 *
Para.				
Area, cm^2^	0.78 (0.43, 1.13)	<0.001 *	0.38 (0.09, 0.67)	0.012 *
Density, HU	0.21 (−0.35, 0.77)	0.445	0.22 (−0.12, 0.56)	0.189
Ps.				
Area, cm^2^	0.88 (0.31, 1.44)	0.004 *	0.82 (0.31, 1.33)	0.003 *
Density, HU	0.49 (0.04, 0.93)	0.035 *	0.43 (0.15, 0.71)	0.005 *
**Muscle function**				
Handgrip strength, kg	0.74 (0.21, 1.26)	0.008 *	0.61 (0.30, 0.92)	<0.001 *

Abbreviations: *B*, regression coefficient; CI, confidence interval; Para., paraspinal muscle; Ps., psoas muscle; SAT, subcutaneous adipose tissue; VAT, visceral adipose tissue. * denotes significant *p*-value.

**Table 6:** The association between change of body composition and change of PSA in the exercise group.

Parameters	6th Month—Baseline		12th Month—Baseline	
	*B* (95% CI)	*p*-Value	*B* (95% CI)	*p*-Value
**DXA parameters**				
Fat, per 100 g				
Arm	−156.44 (−253.26, −59.61)	0.003 *	−80.07 (−135.61, −24.53)	0.007 *
Trunk	−9.38 (−21.31, 2.55)	0.118	−10.84 (−23.98, 2.30)	0.101
Leg	−57.56 (−99.92, −15.20)	0.010 *	−33.97 (−55.69, −12.24)	0.004 *
Total	−9.95 (−51.27, 31.38)	0.626	−7.03 (−46.33, 32.27)	0.715
Lean, per 100 g				
Arm	−107.64 (−205.94, −9.33)	0.033 *	−79.41 (−134.81, −24.01)	0.007 *
Trunk	0.57 (−44.44, 45.58)	0.980	−0.16 (−52.49, 52.17)	0.995
Leg	−54.72 (−99.20, −10.24)	0.018 *	−26.82 (−46.61, −7.03)	0.010 *
Total	3.80 (−18.63, 26.22)	0.731	1.69 (−25.60, 28.98)	0.899
ASMI, kg/m^2^	−555.51 (−1040.85, −70.18)	0.026 *	−564.60 (−996.65, −132.54)	0.013 *
**CT parameters**				
SAT				
Area, cm^2^	−7.32 (−14.45, −0.20)	0.044 *	−7.82 (−16.94, 1.29)	0.089
Density, HU	6.59 (−13.64, 26.83)	0.510	8.58 (−9.65, 26.80)	0.340
VAT				
Area, cm^2^	−2.11 (−8.23, 4.02)	0.487	−3.18 (−9.81, 3.44)	0.331
Density, HU	16.02 (−9.74, 41.77)	0.213	14.42 (−7.97, 36.82)	0.196
Para.				
Area, cm^2^	−54.96 (−93.00, −16.92)	0.006 *	−60.98 (−102.85, −19.11)	0.006 *
Density, HU	−5.58 (−58.26, 47.11)	0.830	−2.51 (−53.96, 48.94)	0.920
Ps.				
Area, cm^2^	−63.06 (−119.90, −6.22)	0.031 *	−86.10 (−168.13, −4.08)	0.040 *
Density, HU	−18.63 (−63.79, 26.52)	0.405	−20.45 (−68.81, 27.90)	0.391
**Muscle function**				
Handgrip strength, kg	−27.52 (−82.94, 27.90)	0.318	−46.34 (−101.36, 8.68)	0.095

Abbreviations: *B*, regression coefficient; CI, confidence interval; Para., paraspinal muscle; Ps., psoas muscle; SAT, subcutaneous adipose tissue; VAT, visceral adipose tissue. * denotes significant *p*-value.

## Data Availability

Data of the study is available upon reasonable requests to the corresponding author.

## Supplementary Materials

The following supporting information can be downloaded at: https://www.mdpi.com/article/10.3390/nu14235088/s1, Supplementary Table S1: The association between the change of body composition (from baseline to the 6th month) and the change of prognostic nutrition index and PSA (from baseline to the 12th month) in the exercise group.
