# Supplementary material for: The Impact of Exercise on Improving Body Composition and PSA in High-Risk Prostate Cancer Patients on Androgen-Deprivation Therapy
Source: Nutrients. 2022 Nov 30;14(23):5088. doi: 10.3390/nu14235088 (PMC9738737; doi:10.3390/nu14235088)
Supplement: Supplementary file 1 [file nutrients-14-05088-s001.zip › nutrients-2023740-supplementary.pdf]

Supplementary Table S1. The association between the change of body composition (from baseline to the 6th month) and the change of prognostic nutrition index and PSA (from baseline to the 12th month) in the exercise group.

**Parameters (6th month – baseline)**	**12th month - baseline**			
	Prognostic nutrition index		PSA, ng/mL	
	***B* (95% CI)**	***P* value**	***B* (95% CI)**	***P* value**
**<u>DXA parameters</u>**				
Fat, per 100 g				
Arm	0.93 (0.12, 1.75)	0.026*	-189.83 (-300.41, -79.26)	0.002*
Trunk	0.12 (0.03, 0.20)	0.009*	-11.60 (-26.25, 3.05)	0.115
Leg	0.56 (0.25, 0.88)	0.001*	-76.17 (-128.55, -23.79)	0.006*
Total	0.41 (0.15, 0.68)	0.004*	-9.47 (-56.04, 37.10)	0.678
Lean, per 100 g				
Arm	1.08 (0.38, 1.77)	0.004*	-121.23 (-231.73, -10.73)	0.033*
Trunk	-0.21 (-0.55, 0.12)	0.202	3.48 (-49.96, 56.93)	0.894
Leg	0.57 (0.26, 0.89)	0.001*	-82.49 (-135.43, -29.56)	0.004*
Total	-0.17 (-0.32, -0.01)	0.036*	4.99 (-20.59, 30.58)	0.691
ASMI, kg/m^2	5.83 (2.49, 9.16)	0.001*	-805.48 (-1385.65, -225.31)	0.009*
**<u>CT parameters</u>**				
SAT				
Area, cm^2	0.08 (0.03, 0.13)	0.005*	-10.78 (-19.66, -1.89)	0.019*
Density, HU	-0.16 (-0.30, -0.02)	0.026*	9.05 (-14.08, 32.17)	0.427
VAT				
Area, cm^2	0.04 (-0.01, 0.09)	0.083	-1.91 (-9.67, 5.85)	0.617
Density, HU	-0.29 (-0.45, -0.13)	0.001*	19.23 (-10.08, 48.55)	0.188
Para.				
Area, cm^2	0.56 (0.33, 0.79)	<0.001*	-74.91 (-116.92, -32.90)	0.001*
Density, HU	0.18 (-0.21, 0.57)	0.358	-0.43 (-58.50, 57.64)	0.988
Ps.				
Area, cm^2	0.39 (-0.05, 0.84)	0.080	-80.14 (-144.12, -16.15)	0.016*
Density, HU	0.30 (-0.02, 0.62)	0.061	-15.51 (-65.45, 34.43)	0.528
**<u>Muscle function</u>**				
Handgrip strength, kg	0.47 (0.09, 0.85)	0.018*	-32.98 (-94.15, 28.19)	0.277

Abbreviations: *B*, regression coefficient; CI, confidence interval; Para., paraspinal muscle; Ps., psoas muscle; PSA, prostate-specific antigen; SAT, subcutaneous adipose tissue; VAT, visceral adipose tissue.
* denotes significant p-value.
